# Supplementary material for: MetaRibo-Seq measures translation in microbiomes
Source: Nat Commun. 2020 Jun 29;11:3268. doi: 10.1038/s41467-020-17081-z (PMC7324362; doi:10.1038/s41467-020-17081-z)
Supplement: Supplementary file 10 — Supplementary Data 7 [file 41467_2020_17081_MOESM10_ESM.zip › File2/Confidence_High_Taxonomy/64303_out.krona.html]

Javascript must be enabled to view this page.

members
magnitude
magnitudeUnassigned
count
unassigned
taxon
rank

64303\_out

3

superkingdom
2
3

phylum
3
1239

186801
3
class

order
186802
3

family
3
541000

216851
3
genus

3
853
species

SRS018623\_contig\_number\_contig-100\_13198.60721SRS050998\_contig\_number\_contig-100\_4297.105986SRS1041091\_contig\_number\_contig-100\_5472.101062
